# Maternal and perinatal health research priorities beyond 2015: an international survey and prioritization exercise

**DOI:** 10.1186/1742-4755-11-61

**Published:** 2014-08-07

**Authors:** Joao Paulo Souza, Mariana Widmer, Ahmet Metin Gülmezoglu, Theresa Anne Lawrie, Ebunoluwa Aderonke Adejuyigbe, Guillermo Carroli, Caroline Crowther, Sheena M Currie, Therese Dowswell, Justus Hofmeyr, Tina Lavender, Joy Lawn, Silke Mader, Francisco Eulógio Martinez, Kidza Mugerwa, Zahida Qureshi, Maria Asuncion Silvestre, Hora Soltani, Maria Regina Torloni, Eleni Z Tsigas, Zoe Vowles, Léopold Ouedraogo, Suzanne Serruya, Jamela Al-Raiby, Narimah Awin, Hiromi Obara, Matthews Mathai, Rajiv Bahl, José Martines, Bela Ganatra, Sharon Jelena Phillips, Brooke Ronald Johnson, Joshua P Vogel, Olufemi T Oladapo, Marleen Temmerman

**Affiliations:** 1 UNDP/UNFPA/UNICEF/WHO/World Bank Special Programme of Research, Development and Research Training in Human Reproduction (HRP), Department of Reproductive Health and Research, World Health Organization, Geneva, Switzerland; 2 Department of Social Medicine, Ribeirão Preto Medical School, University of São Paulo, Ribeirão Preto, São Paulo, Brazil; 3 Royal United Hospital, Bath, UK; 4 Faculty of Clinical Sciences, Obafemi Awolowo University, Ile-Ife, Osun State, Nigeria; 5 Centro Rosarino de Estudios Perinatales (CREP), Rosario, Santa Fé, Argentina; 6 Liggins Institute, The University of Auckland, Grafton, Auckland, New Zealand; 7 Maternal and Child Health Integrated Program (MCHIP), Johns Hopkins University Program for International Education in Reproductive Health (JHPIEGO), Baltimore, USA; 8 Cochrane Pregnancy and Childbirth Group, Liverpool University, Liverpool, UK; 9 Effective Care Research Unit, University of the Witwatersrand / University of Fort Hare / Eastern Cape Department of Health, Amalinda Drive, East London, Eastern Cape, South Africa; 10 School of Nursing, Midwifery & Social Work, University of Manchester, Manchester, UK; 11 MARCH (Maternal, Reproductive and Child Health) Center, London School of Hygiene and Tropical Medicine, London, UK; 12 European Foundation for the Care of Newborn Infants, Munich, Germany; 13 Department of Pediatrics, Ribeirão Preto Medical School, University of São Paulo, Ribeirão Preto, São Paulo, Brazil; 14 Department of Obstetrics and Gynecology, School of Medicine, College of Health Sciences, Makerere University, Kampala, Uganda; 15 Department of Obstetrics & Gynaecology, University of Nairobi, Nairobi, Kenya; 16 Kalusugan ng Mag-Ina (Health of Mother and Child), Inc, New Manila, Quezon City, Philippines; 17 Health and Social Care Research Centre, Sheffield Hallam University, Sheffiled, UK; 18 Department of Obstetrics, School of Medicine of São Paulo, São Paulo Federal University, São Paulo, Brazil; 19 Preeclampsia Foundation, Melbourne, Florida, USA; 20 International Confederation of Midwives, The Hague, The Netherlands; 21 WHO Regional Office for Africa, Brazzavile, Republic of the Congo; 22 Latin American Center for Perinatology, Women and Reproductive Health, (CLAP/WR), WHO Regional Office for the Americas, Montevideo, Uruguay; 23 WHO Regional Office for the Eastern Mediterranean, Cairo, Egypt; 24 WHO Regional Office for South East Asia, New Delhi, India; 25 WHO Regional Office for the Western Pacific, Manila, Philippines; 26 Department of Maternal, Newborn, Child and Adolescent Health, World Health Organization, Geneva, Switzerland; 27 School of Population Health, Faculty of Medicine, Dentistry and Health Sciences, University of Western Australia, Perth, Australia

**Keywords:** Research priorities, CHNRI, Maternal and perinatal health

## Abstract

**Background:**

Maternal mortality has declined by nearly half since 1990, but over a quarter million women still die every year of causes related to pregnancy and childbirth. Maternal-health related targets are falling short of the 2015 Millennium Development Goals and a post-2015 Development Agenda is emerging. In connection with this, setting global research priorities for the next decade is now required.

**Methods:**

We adapted the methods of the Child Health and Nutrition Research Initiative (CHNRI) to identify and set global research priorities for maternal and perinatal health for the period 2015 to 2025. Priority research questions were received from various international stakeholders constituting a large reference group, and consolidated into a final list of research questions by a technical working group. Questions on this list were then scored by the reference working group according to five independent and equally weighted criteria. Normalized research priority scores (NRPS) were calculated, and research priority questions were ranked accordingly.

**Results:**

A list of 190 priority research questions for improving maternal and perinatal health was scored by 140 stakeholders. Most priority research questions (89%) were concerned with the evaluation of implementation and delivery of existing interventions, with research subthemes frequently concerned with training and/or awareness interventions (11%), and access to interventions and/or services (14%). Twenty-one questions (11%) involved the discovery of new interventions or technologies.

**Conclusions:**

Key research priorities in maternal and perinatal health were identified. The resulting ranked list of research questions provides a valuable resource for health research investors, researchers and other stakeholders. We are hopeful that this exercise will inform the post-2015 Development Agenda and assist donors, research-policy decision makers and researchers to invest in research that will ultimately make the most significant difference in the lives of mothers and babies.

## Background

In 2000, heads of States and Governments gathered at the United Nations General Assembly and agreed to put in place an international effort to eradicate extreme poverty and promote human development. The 2000 Millennium Summit resulted in a series of time-bound targets (the Millennium Development Goals - MDGs) which include improving maternal health and reducing child mortality by 2015. Improving maternal health, considered a crucial element to combat poverty and underdevelopment on a global scale, consisted of two components: reducing maternal mortality, and achieving universal access to reproductive health services [1].

Maternal mortality has declined by nearly half since 1990, but this progress falls short of the MDG target and over a quarter million women still die every year of causes related to pregnancy and childbirth [2,3]. Maternal health-related indicators are among the worst performing in the MDG effort; and only a small number of countries will reach their maternal mortality targets by 2015 [3]. Despite this relatively slow progress, it is widely believed that the interventions needed to reduce maternal mortality ratios (MMR) to less than 50 deaths per 100,000 live births per year globally, already exist [4]. Obstacles to implementing effective interventions and disseminating knowledge delay progress, particularly in the least developed countries and most vulnerable populations. With the deadline for the MDGs approaching, the international community is currently mobilizing to develop plans for the post-MDG era [4]. As a part of this global effort, we conducted an international survey and prioritization exercise to identify key research priorities that could accelerate improvement in maternal and perinatal health from 2015 to 2025.

The Child Health and Nutrition Research Initiative (CHNRI) has developed a method to assist policy makers, donors and stakeholders in understanding the potential of different research avenues to contribute to reducing the burden of disease and disability [5]. This method is participatory, identifies weaknesses and strengths of proposed research options and enables transparent prioritization for research investment [5-7]. The CHNRI methods have been adapted and applied at national and international levels in various fields and the World Health Organization (WHO) has used these methods for several previous research prioritization exercises [8-11]. In this paper, we present the results of the WHO research prioritization exercise using adapted CHNRI methods to identify global research priorities for maternal and perinatal health.

## Methods

**Figure 1:**
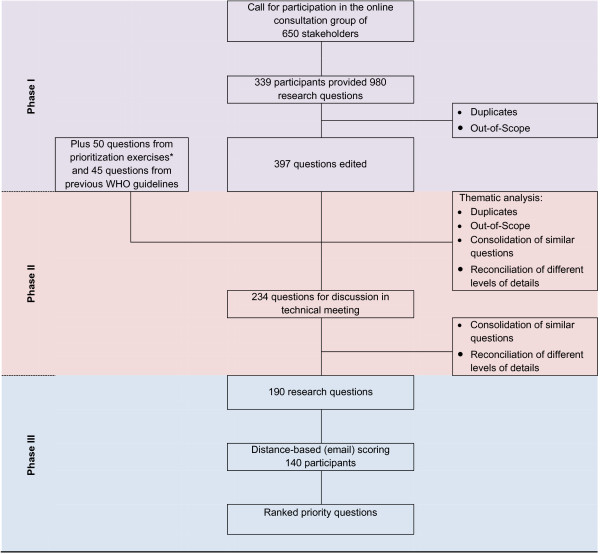
Study and analysis flow.

The CHNRI methods have been previously published together with detailed guidelines for implementation [5]. The goal of our priority setting exercise was to identify research questions with the potential to have an impact on maternal and perinatal health indicators between 2015 and 2025. In this context, maternal health’ relates to conditions affecting women during pregnancy, childbirth/abortion and up to six weeks postpartum/post-abortion, and ‘perinatal health’ relates to conditions affecting offspring from the time of fetal viability to the first 28 postnatal days. This process was managed by the WHO and implemented in three phases: (1) the generation and collection of research questions, (2) thematic analysis and consolidation of research questions, and (3) prioritization of research questions using a scoring system based on five criteria. Figure 1 illustrates this process.

Phase I was initiated by establishing a reference group of researchers, health care providers, program managers, and other stakeholders (including representatives of consumer groups and donors). An invitation was sent to a large number of active researchers in the field of maternal and perinatal health, identified through bibliographic metrics and other information available in the “BiomedExperts” database. This database includes over 400,000 registered members and 1.8 million pre-generated profiles of life science researchers (http://www.biomedexperts.com/). Potential participants were identified in the BiomedExperts database using a pre-specified search strategy available in Appendix 1. The identification of researchers was stratified to ensure participation of researchers from both developed and developing countries. In addition, invitations were also sent to program managers and policymakers identified in contact lists of WHO and partner organizations (e.g. the United States Agency for International Development (USAID) Maternal and Child Health Integrated Program (MCHIP)). Those who responded positively to the invitation became members of the reference group. All members of the reference group were invited to provide three research questions in seven domains: obstetric haemorrhage, hypertensive disorders of pregnancy (HDP), maternal sepsis, abortion, difficult/obstructed labour, preterm birth, and stillbirth. Maternal and perinatal health research questions identified through other processes (including a USAID priority setting exercise, published WHO guidelines, and a previous WHO CHNRI intrapartum priority setting exercise) were also included in the index list of research questions [12-16].

In Phase II, this long list of questions was independently assessed by two researchers (MW and SJP) for identification of duplicate questions. Questions that were out of scope (i.e. not pertaining to any of the previously mentioned domains), or that were too broad to be considered research questions (e.g. “research to reduce maternal mortality”, “develop and test interventions for reducing postpartum haemorrhage”), or that were considered epidemiological (non-intervention) research, were excluded. This process was reviewed by a third researcher (JPS), who resolved discrepancies. A reduced list of questions was then submitted to thematic analysis. The thematic analysis consisted of grouping similar questions together to identify research themes and sub-themes. This allowed us to identify additional duplicates and out-of-scope questions. Questions were edited for clarity and similar questions were merged. During this process, we aimed to achieve a certain level of detail compatible with the concept of “research avenues” (i.e. a research question that is not too broad, neither too specific, and could be answered through a set of individual research projects); hence, very detailed and specific questions were made more general. This process resulted in a refined list of questions for the technical consultation meeting held in Geneva in April 2013. The large majority of the participants in this technical consultation was selected from amongst the reference group and composed the technical working group. This technical working group consisted of a diverse group of 22 participants that included clinical specialists, researchers, program managers, WHO officers, donor and consumer representatives, and other stakeholders. During this technical consultation, the product of the thematic analysis was reviewed, new questions were developed where omissions/gaps were identified, and similar questions were further consolidated.

Phase III consisted of scoring the final list of research questions. To reduce bias due to participant fatigue, we prepared six spreadsheets that differed in the order in which the research questions were presented. Each member of the reference group received one of these electronic spreadsheets via e-mail, accompanied by a score sheet consisting of five criteria to be used for scoring the questions. These five criteria included answerability, effectiveness, deliverability, maximum potential for disease burden reduction, and equity (Table 1). They are described in detail in the CHNRI guidelines [5]. The participants were instructed to score the questions one criterion at a time using a binary score system (1: Yes, 0: No). If they were not sure, did not know, or were not able to make a judgment, they were asked to leave the question blank. The completed spreadsheets, when returned by the participants, were integrated into a database.

**Table 1:** Scoring criteria for setting research priorities

**1.**	**Answerability**	The research question can be ethically answered.
**2.**	**Effectiveness**	The new knowledge is likely to result in an effective intervention or program.
**3.**	**Deliverability**	The intervention or program will be deliverable, acceptable and affordable.
**4.**	**Potential impact**	The intervention or program has the potential to substantially reduce maternal and perinatal mortality, morbidity and long term disabilities.
**5.**	**Equity**	The intervention or program will reach the most vulnerable groups.

A research priority score was generated for each question by summing up the scores attributed to each criterion. No special weighting of criteria was applied. Thus, for each individual respondent, each research question could have a priority score ranging from 0 to 5. The overall research priority score was computed as the sum of all individual research priority scores. For each question, the overall research priority score was normalized (i.e. considering all questions, the overall research priority score for the question was subtracted by the minimum research priority score among all questions, and divided by the range: (x - min)/(max - min)). The normalized research priority score (NRPS) was analyzed and the cut-off point, enabling identification of the upper quartile (questions with the highest normalized research priority scores), was determined. Online Google® forms were used to capture online data from the reference group and Microsoft Excel (2010) spreadsheets were used to score and analyze the responses provided.

## Results

**Figure 2:**
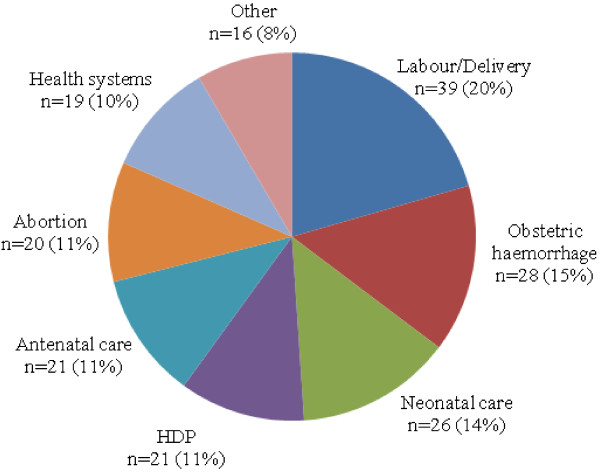
Priority research questions (N = 190) by theme.

A total of 650 stakeholders responded positively to our initial invitation to participate in this exercise and were included in the reference group. Of these, 339 participants (52%) provided 980 research ideas or questions; these were considered together with 95 research questions generated through other recent research prioritization processes. Participants from 67 countries provided research ideas or questions (22 developed countries contributed with 44% of participants, 45 developing countries contributed with 56% of participants). Researchers (37%; 125/339), Clinician physicians (27%; 92/339); program managers and policy makers (20%; 67/339) were the main providers of research questions. Midwives, donor representatives, consumers and other stakeholders provided also research questions. After exclusion of duplicates, thematic analysis and editing, 234 questions were discussed by the technical working group, working closely with the WHO management team, at the technical consultation meeting held in Geneva. The technical consultation produced a consolidated list with 190 research questions, which was sent to the reference group for scoring. A total of 140 participants (22%) of the reference group scored the questions and returned completed spreadsheets, which were integrated into one database.The distribution of research questions generated per theme is shown in Figure 2. Overall, most of the research questions (89%) address the implementation of existing interventions or knowledge (delivery/implementation research); 21 questions (11%) address research to discover new interventions or technologies (discovery research).

Additional file 1 contains the database with all research priority questions and criteria scores. Normalized scores for the 190 questions ranged from 0 to 100. Questions with a NRPS of 76 and above formed part of the upper quartile of highest ranked questions (56 in total). These were fairly evenly distributed, with each of the main themes attracting six to nine questions in the upper quartile (Table 2).

**Table 2:** Breakdown of research question flow by themes

**Research themes and common sub-themes**	**Total questions (N = 190)**	Top-ranked questions* (n = 56)	**Top-twenty questions (n = 20)**
**Labour and delivery:** Preterm birth, difficult/obstructed labour, fetal monitoring, the partograph, training and/or awareness, caesarean section, management of the third stage, induction of labour	39 (21%)	6 (11%)	2 (10%)
**Obstetric haemorrhage:** Misoprostol access, uterotonics (type, dose, route), screening and detection, training and/or awareness, care quality, blood transfusions, management of the third stage	28 (15%)	8 (14%)	3 (15%)
**Hypertensive disorders of pregnancy:** Screening and detection, magnesium sulphate, biochemical markers, anticonvulsants, antihypertensive agents, training and/or awareness, prevention	21 (11%)	8 (14%)	0 (0%)
**Abortion:** Post-abortion contraception, misoprostol access, post-abortion follow-up, abortion in restricted settings, second trimester abortion, training and/or awareness	20 (11%)	8 (14%)	5 (25%)
**Antenatal care:** Screening and detection (including impaired fetal growth, infection, preterm birth, anaemia), ultrasound access, nutrition, malaria, diabetes care	21 (11%)	6 (11%)	2 (10%)
**Health systems:** Transport and communication, service quality, emergency services, mobile community health services, supervision and mentoring, service utilization, monitoring and audits	19 (10%)	8 (14%)	4 (20%)
**Neonatal care:** Neonatal resuscitation, hypoxic ischaemic encephalopathy, screening and detection, kangaroo mother care, cord care, care of preterm neonates, training and/or awareness	26 (14%)	9 (16%)	3 (15%)
**Other:** Puerperal sepsis, postnatal care, improving attitudes/behaviour of healthcare workers	16 (8%)	2 (4%)	1 (5%)

*Upper quartile questions with a normalized research priority score (NRPS) of 76 and above.

The top 20 highest ranked questions overall are presented in Table 3. Abortion research makes up 25% (n = 5) of the top 20 list with other major themes including health systems research (n = 4), obstetric haemorrhage (n = 3), neonatal care (n = 3), and labour/delivery (n = 2). Training and/or awareness interventions comprise 30% (n = 6) of the sub-themes of these top 20 research priorities.

**Table 3:** Top 20 (highest scoring) research priority questions to improve maternal and/or perinatal health outcomes between 2015 and 2025

**Research question**	**NRPS**	**Theme**
Evaluate the effectiveness of interventions (e.g. counselling or incentives, or home visits) to increase post-abortion contraception uptake and continuance, and reduce repeat abortion	100	Abortion
Evaluate the effectiveness and costs of strategies to improve the quality and utilization of maternity services (e.g. maternity waiting homes, improved communication via mobile phones, community awareness strategies) to improve early detection and management of antenatal and intrapartum complications	95	Health systems
Develop and evaluate strategies for locally appropriate transport, communication and referral systems for obstetric and newborn emergencies	94	Health systems
Evaluate the effectiveness and cost of strategies to prevent, detect and treat causes of anaemia in pregnancy (e.g. malaria, occult bleeding disorders, nutritional deficiencies)	93	Antenatal care
Evaluate the effectiveness and cost of training interventions for frontline healthcare workers (paramedics, doctors, CHWs, midwives, nurses) to diagnose, manage and refer women with obstetric haemorrhage	92	Obstetric haemorrhage
Evaluate the effectiveness and cost of a package of community level interventions for preterm babies (e.g. implementing and providing guidelines for kangaroo mother care, home visits by CHWs, infection prevention strategies)	92	Neonatal care
Evaluate the effectiveness of integrating abortion services into existing family planning services	91	Abortion
Evaluate the effectiveness and cost of training frontline healthcare workers, including nurses, midwives and community health workers, to detect and treat neonatal sepsis (or to provide pre-referral treatment only)	90	Neonatal care
Develop and evaluate community-based awareness programs to reduce unwanted pregnancies and encourage women to seek help early	89	Abortion
Evaluate the effectiveness and cost of training interventions for skilled birth attendants to gain and maintain competence in the management of obstructed labour, and assisted delivery techniques	88	Labour and delivery
Evaluate the effectiveness and cost of training skilled birth attendants in intrapartum fetal monitoring and neonatal resuscitation for reducing stillbirths and deaths/disability due to perinatal asphyxia	88	Neonatal care
Evaluate the effectiveness and cost of a package of interventions for the prevention, early detection and treatment of puerperal sepsis (e.g. sterile birth kits, access to antibiotics, automated thermometers)	88	Other (puerperal sepsis)
Evaluate the effectiveness and cost of a package of mobile service interventions delivered at community level, including mobile clinics and home-based care, on maternal and perinatal health outcomes	87	Health systems
Evaluate the effectiveness, safety and timing of the initiation of post-abortion contraception (hormonal and IUDs) with respect to abortion outcomes, contraceptive effectiveness, uptake, continuance, and repeat abortions	87	Abortion
Develop and evaluate the effectiveness and cost of strategies to improve access of women with obstetric haemorrhage to blood and blood replacement products in settings without transport capabilities	87	Obstetric haemorrhage
Develop and evaluate the effectiveness of strategies to increase access of women to misoprostol at community level where oxytocin is not available/feasible, by dispensing it antenatally as part of a birthing kit, or at the time of delivery via the attending CHW or nurse/midwife, to prevent and treat PPH	87	Obstetric haemorrhage
Develop and evaluate strategies to increase appropriate use of the partograph, including decision-making and action, to improve maternal and perinatal health outcomes	85	Labour and delivery
Evaluate the effectiveness and cost of strategies, including task-shifting, to increase access of women to high quality post-abortion care to improve early detection of complications	85	Abortion
Assess the effectiveness and cost of implementing a package of screening and treating syphilis and HIV in women of reproductive age to improve maternal and perinatal health outcomes.	85	Antenatal care
Develop and evaluate a health systems package for effective task shifting for the management of obstetric emergencies, including protocols, supervisory systems, and metrics	83	Health systems

NRPS: Normalized research priority score. This was performed by subtracting the minimum research priority score from the index question score, and dividing by the NRPS range, i.e. (x - min)/(max - min).

Hypertensive disorders of pregnancy (HDP) is not represented in the top-20 list but comprises 14% of the questions in the upper quartile. Similarly, none of the upper quartile questions were ‘discovery’ questions. The five highest scoring discovery questions with their ranking can be found in Table 4.

**Table 4:** Top five priority research questions addressing new health interventions (discovery questions) to improve maternal and perinatal health

**Research question**	**NRPS**	**Theme**
Discover new formulations of uterotonics (e.g. low-cost, simple to use, non-invasive, heat-stable) to prevent and treat PPH and improve maternal health outcomes.	72	Obstetric haemorrhage
Discover and evaluate a standardised method of measuring blood loss to improve the detection and management of PPH, to improve maternal health outcomes.	70	Obstetric haemorrhage
Discover new technologies/screening tools for the detection of anaemia in pregnancy to improve maternal and perinatal health outcomes.	66	Antenatal care
Discover and evaluate new methods/technologies to prevent and treat obstetric haemorrhage and improve maternal health outcomes.	55	Obstetric haemorrhage
Discover and evaluate new pharmaceutical treatments for eclampsia to improve maternal and perinatal outcomes.	48	HDP

HDP: hypertensive disorders of pregnancy.

## Discussion

The current exercise led to the identification of research questions that are mostly related to implementation of existing interventions and the development of simplified, more cost-effective versions of existing interventions (e.g. oxytocin and misoprostol for PPH prevention and treatment or magnesium sulfate for eclampsia). A limited number of questions related to the discovery of new solutions were proposed (e.g. new uterotonics for PPH or new ways to identify PPH). The opinions expressed by the participants through their input in this process appear to corroborate the notion that the biggest challenge in current maternal and perinatal health is to increase the outreach of existing effective solutions for those who need them most.

These results represent the consensus view of a large number of researchers, policymakers and other stakeholders internationally, and provide the essential routes to action to eliminate preventable maternal deaths by 2025. CHNRI methods aim to standardize and make more transparent the highly complex process of setting research priorities. Overall, these methods are perceived as robust, but there are some concerns related to the ability of these methods to identify all relevant research ideas. In general, most of the previous CHNRI exercises have relied on relatively small reference groups (in general, < 100 participants). Small reference groups are more likely to be biased by various elements of their composition and more dominant individuals within the group. In the present exercise, we tried to overcome these potential weaknesses by increasing the number of participants and using distance-based methods for collecting and scoring questions. Thus, this became the largest exercise for prioritizing research questions in maternal and perinatal health to date, involving a large number of experts and stakeholders, and with a very large number of questions generated. However, some limitations should be noted. Having a large group of experts and stakeholders was a positive factor because it ensured broader representation and a very large pool of research questions. However, managing a large number of questions was technically challenging for all involved, particularly at list preparation (phase I) and scoring (phase II) stages. Given the broad scope of this exercise (maternal and perinatal health) and the long list of priority questions to be scored, participant fatigue and time constraints were of concern. For the set of 190 questions that was sent to the reference group, each respondent had to attribute a total of 950 scores (190 questions times five criteria), which was a very time-intensive task. Anecdotal accounts suggest that the time required to complete the task was approximately four hours, however, some respondents reported taking eight hours or more. This may explain the substantial drop in response rate during phase III. Developing and testing simplified versions of the CHNRI process could be explored as a way to strike a balance between the necessary methodological rigor and practical implementation. Another limitation of this method is an apparent trend towards prioritizing implementation research questions over discovery questions. This was observed in this exercise and it has been a feature of other CHNRI prioritization exercises; for example, in a similar exercise for setting global mental health research priorities, new interventions and technologies comprised seven out of the ten lowest-scoring priorities, and none of the top ten scoring priorities [17]. A possible explanation for this could be that discovery questions tend to be more innovative and inherently riskier in terms of research investment while implementation research question seem to respond concretely to immediate needs. Consensus-driven processes are conservative by nature and as a result, research questions that seem “safer” (e.g. implementation research questions) could be more appealing to large consultation groups. An additional explanation is that some stakeholders are simply not aware of some of the new technologies that are in earlier phases of development. Thus, when asked to list research questions, they could focus on the technologies that they know about. One may question the validity of the CHNRI methodology to assess research that is at the discovery stage as criteria such as effectiveness, deliverability and equity are less relevant at the discovery stage (though they may become more relevant later, depending on the outcome of the primary research). The method thus tends to systematically attribute lower scores to discovery research. As future advances in the field would depend on discovery research, in future exercises, exploring ways of counter-balancing this trend would be advisable; for example, conducting dedicated modules with more relevant criteria (such as safety or innovation) on discovery questions could avoid competition between different types of research questions.

The high ranking of priority research for abortion and obstetric haemorrhage in this exercise reflects the substantial contribution of these aspects to maternal mortality and morbidity rates. Recurring sub-themes for abortion research were mainly concerned with training and/or awareness interventions, access to abortion, and post-abortion care and contraception. Similarly, for obstetric haemorrhage, training and/or awareness interventions, and access to existing uterotonics were common sub-themes. Given that both abortion and haemorrhage are the most avoidable causes of maternal morbidity and mortality, it is no surprise that priority questions focused on the implementation/delivery of known effective interventions. Another recurring theme is research to address cost-effectiveness knowledge gaps, which denotes the importance of sustainability of new health technologies. Health systems research had the second most research questions in the top-20 list, illustrating the need for effective health systems to enable effective service delivery. This emphasis on implementation research and health system research suggest that the international community is keen in overcoming barriers for using what is already available rather than developing new technologies that may be not used due to the same reasons that are currently preventing the use of existing health technology. As emphasis shift towards light technologies (i.e. focus on work processes and system thinking) further methodological developments to ensure proper evidence generation are needed.

In this exercise, members of the reference group were drawn from a diverse and widely representative group of stakeholders, who contributed to, and scored, questions according to well-defined criteria; therefore we consider this exercise to reflect the priorities for global research in maternal and perinatal health going forward. Unlike most other priority setting exercises using the CHNRI approach, we did not weight the criteria used for setting priorities, but have published the complete list of research questions with individual criteria scores. This should enable stakeholders to generate customized research priorities according to their own weightings (e.g. a donor agency may wish to promote research questions that contribute more to health equity, or a governmental foundation may wish to tackle research questions that will have the largest impact on the disease burden), and risk management preferences.

## Conclusions

Key research priorities in maternal and perinatal health were identified. The resulting ranked list of research questions provides a valuable resource for health research investors, researchers and other stakeholders. We are hopeful that this exercise will inform the post-2015 Development Agenda and assist donors, research-policy decision makers and researchers to invest in research that will ultimately make the most significant difference in the lives of mothers and babies.

## Appendix 1

### BiomedExperts search strategy

BiomedExperts is a free online service for the life sciences community to connect, network, communicate and collaborate. BiomedExperts contains the research profiles of more than 1.8 million life science researchers, representing over 26 million connections from over 2,700 institutions in more than 160 countries. These profiles were generated from author and co-author information from 18 million publications published in over 20,000 journals.

### Search strategy

Keywords/areas of research searched:

• Caesarean section

• Eclampsia

• Fistula

• Labour complications

• Maternal mortality

• Postpartum haemorrhage

• Preeclampsia

• Pregnancy induced hypertension

• Preterm births

• Sepsis

• Still births

### Identification of researchers

For each of the above areas, we chose the first 20 authors at “Global” level that published the highest number of articles related to that area, the first 10 authors at coauthors level 1 (“your coauthors”) and the first 10 authors at coauthors level 2 (“coauthors of your coauthors”). The identification of researchers was stratified to ensure participation of researchers from both developed and developing countries.

## Abbreviations

MDG: Millennium development goals; MMR: Maternal mortality ratios; CHNRI: Child health and nutrition research initiative; WHO: World health organization; USAID: The United States agency for international development; MCHIP: Maternal and child health integrated program; HDP: Hypertensive disorders of pregnancy; NRPS: Normalized research priority score.

## Competing interests

The authors declare that they have no competing interests.

## Authors’ contributions

AMG, JPS, MW, RB and JM, conceived the idea for the exercise. JPS and MW led the international survey and prioritization exercise. JPS led the writing of the paper with contributions from all authors. JPS and TAL prepared tables and figures. All authors read and approved this manuscript.

## Supplementary Material

Additional file 1 Final list of research questions. Click here for file
